# Global mapping of physical activity promotion strategies in Fibromyalgia- A comprehensive methodological scoping review protocol

**DOI:** 10.1016/j.mex.2025.103234

**Published:** 2025-02-25

**Authors:** Saumya Srivastava, Aishwarya Nair, Dhanesh Kumar K U, Rakesh Krishna Kovela, Vaishali K, Ashwani Verma, Mukesh Kumar Sinha

**Affiliations:** a Nitte Institute of Physiotherapy, NITTE (Deemed to be University), Deralakatte, Mangalore, Karnataka 575018, India; b Department of Physiotherapy, Manipal College of Health Professions, Manipal Academy of Higher Education, Manipal, Karnataka 576104, India; c Project Coordinator- Data Analytics and Review Mechanism, United Nations Development Programme, New Delhi, India

**Keywords:** Fatigue, Pain, Quality of life, Exercise tolerance, Pain syndrome, Fibromyalgia, Exercise, Physical activity promotion, Scoping Review Method.

## Abstract

Fibromyalgia Syndrome (FMS) is a chronic, broad, diffuse, and non-inflammatory musculoskeletal condition. Several studies were conducted on the effects of physical activity (PA) and strategies to promote it in people with FMS. The resulting scoping review will provide an analysis of the strategies being researched and executed. This study outlines the protocol for a scoping review to collect, analyse and compile the promotion strategies for PA in FMS. The proposed protocol will be guided by Arksey and O'Malley's framework and advanced methodology study by Levac et al. The search method will comprise of terms pertaining to FMS, PA, and its promotion. To ascertain relevant studies, predetermined inclusion and exclusion criteria will be used. Studies included must be performed on FMS patients with PA as the intervention with an outcome related to quality of life, pain, and fatigue. Data extraction of the included studies will be done through three investigators using Rayyan software. Preferred Reporting Items for Systematic Reviews and Meta-Analyses extension for Scoping Reviews (PRISMA-ScR) will be used to describe the study

## Graphical abstract

Graphical abstracts are submitted as separate files.

**Figure:**
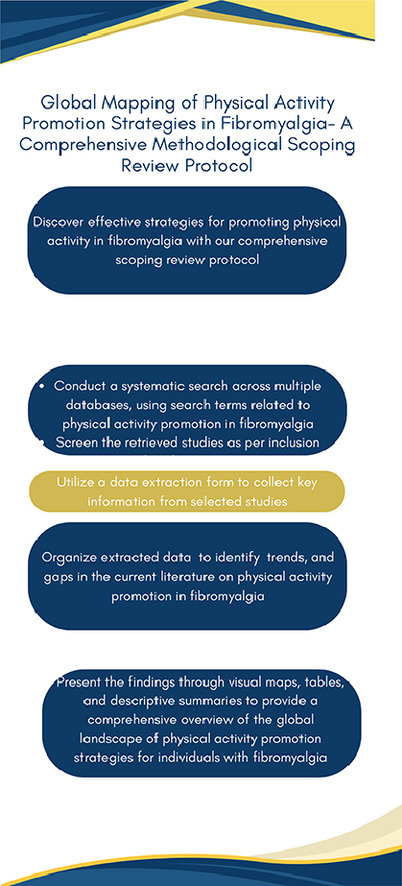

Specifications table

**Table:** 

Subject area:	Medicine and Dentistry
More specific subject area:	musculoskeletal disorder, Pain Science, Physical activity
Name of your protocol:	Scoping Review Protocol
Name of your method:	Scoping Review Method
Reagents/tools:	Not Applicable
Experimental design:	This study outlines the protocol for a scoping review to collect, analyse and compile the promotion strategies for PA in FMS. The proposed protocol will be guided by Arksey and O'Malley's framework and advanced methodology study by Levac et al. The search method will comprise of terms pertaining to FMS, PA, and its promotion. To ascertain relevant studies, predetermined inclusion and exclusion criteria will be used. Studies included must be performed on FMS patients with PA as the intervention with an outcome related to quality of life, pain, and fatigue. Data extraction of the included studies will be done through three investigators using Rayyan software. Preferred Reporting Items for Systematic Reviews and Meta-Analyses extension for Scoping Reviews (PRISMA-ScR) will be used to describe the study.
Trial registration:	Not Applicable
Ethics:	Not Applicable as this manuscript is a Comprehensive Methodology of Scoping Review Protocol
Value of the protocol:	• This study will be one of the foremost articles focusing on a comprehensive methodological mapping, collating, and analysing data on PA promotion strategies in FMS. • The methodology of this research includes an intricate search strategy and a structured, standardized approach adhering to the PRISMA-ScR guidelines conducting extensive abstract and full text article screening with data extraction to ensure inter-reviewer reliability. • This research will aim to provide a systematic and consistent purview of the PA promotion strategies in FMS to exhibit the potential of the knowledge in FMS accurately.

## Background

Fibromyalgia Syndrome (FMS) is a non-inflammatory, chronic, wide, and diffuse musculoskeletal disorder accompanied by fatigue, sleep disturbances, decreased concentration, memory deficits, anxiety, and depression [1]. Inactivity, sedentary lifestyle, and obesity increase the risk of developing FMS. It is the third most common musculoskeletal condition, after low back pain and osteoarthritis, affecting people between the ages of 30 and 35 years, with incidence increasing as they approach middle age. [1,2]

The current evidence for FMS management includes graded physical activity, pharmacotherapy, and behavioral interventions [3]. One of the key therapeutic goals for this health problem is to aid or build up physical activity to enhance function and prevent impairment [3]. In fibromyalgia, physical activity has been demonstrated to improve health outcomes [3]. A study suggests that keeping patients moving is preferable to resting or remaining seated to promote both physical activity and mobility [4]. Physical activity may reduce pain in skeletal muscles by improving the physiological response to muscle microtrauma through increased resilience, restoration, and subsequent adaptation to frequent activity. An array of studies has documented that PA reduces inflammatory and oxidative reactions in the body, influencing anxiety and stress responses in FMS [3,4].

In a study conducted by Larsson et al., the individuals with FMS have described the need and desire to be physically active, along with enlisting the positive experiences after exercise, but have also expressed the fear of performing exercises which reinforces the trepidation of worsening their conditions that would lead to more pain and deficits [5]. Ang DC et al., 2013, conducted a Research to Encourage Exercise in Fibromyalgia (REEF study) that documented the dread of pain and injury as potent inhibitors of participation in long-term physical activity. [6]

The vital aspect of the potency and effectiveness of exercise is maintaining adherence to it, as clearly described by Ang D et al. in 2007.7 The European Alliance of Associations for Rheumatology (EULAR) 2016 recommends the promotion of PA and prioritizes the need to incorporate individually tailored activities for the benefit of FMS individuals. Methods like regular client-therapist interactions, awareness talks, educational sessions, social and emotional support groups, motivational interviewing, and behavioral interventions have been explored to ensure and promote physical activity in individuals with FMS, addictive behavior, and sedentary lifestyles [[7], [8], [9], [10], [11]].

Despite the enormous potential for PA promotion, the literature is still emerging, and the studies are insufficiently systematized, resulting in methodological inconsistencies and significant gaps in our understanding of PA promotion in fibromyalgia. Against this background, this comprehensive methodological scoping review will attempt to explore, map, and collate the available literature for physical activity promotion strategies in Fibromyalgia.

**Description of protocol**.

## Materials and methods

This is a scoping review protocol hence there is no involvement of patients and public. Ethical approval is not entailed as this review will be using previously collected data. The current study will employ the Arksey and O'Malley framework [12], which has been methodologically strengthened by Levac et al. [13]. According to the proposed Arksey and O'Malley structure, this scoping review will be reported in a five-step methodological framework: a) identifying the research question; b) identifying relevant studies; c) study selection d) charting the data; e) collating, summarizing, and reporting the findings [12].

**Stage 1: Identifying the research question**

In this study, we sought to explore the promotion of physical activity (PA) in individuals diagnosed with Fibromyalgia Syndrome (FMS) through a comprehensive scoping review. Our primary aim is twofold: first, to identify and categorize the diverse PA promotion strategies reported within the literature; and second, to evaluate the reported effects of these strategies on critical outcomes such as pain, fatigue, exercise tolerance, and quality of life. FMS is a complex condition characterized by widespread pain and a range of debilitating symptoms, making effective management paramount. To provide a structured analysis of the existing evidence, we will employ the Population, Concept, and Context (PCC) framework established by the Joanna Briggs Institute (JBI) [14]. Under the Population component, our focus will be on individuals with FMS, considering variations in age, severity of symptoms, and demographic factors to ensure a comprehensive understanding of the population at hand. The Concept element of our review will center on the examination of PA promotion strategies, which may encompass various interventions such as educational outreach, structured exercise programs, and community initiatives designed to encourage participation in physical activity. Lastly, the Context dimension will address the settings in which these strategies are implemented, recognizing that factors such as clinical environments, community resources, and home-based settings may significantly influence intervention effectiveness. By systematically reviewing and synthesizing the literature, our scoping review aims to illuminate the existing landscape of PA promotion in FMS, identify gaps in the current research, and ultimately contribute to the development of effective interventions that can enhance the health and quality of life for individuals living with this challenging syndrome (Table 1).

**Table 1:** Eligibility criteria for the studies.

Population	Individuals diagnosed with FMS with Age>18 years of any gender.
Concept	Physical activity (PA) promotion strategies and interventions in individuals with FMS and its effects on health outcomes. (Pain, fatigue, exercise tolerance, and quality of life)
Context	All studies will be reported as per geographical location where individuals with FMS are given PA promotion interventions.

FMS: Fibromyalgia Syndrome; PA: Physical Activity.

**Stage 2: Identifying relevant studies**

This stage, according to Arksey and O'Malley's framework [12], comprises of identifying the available literature. The search will use Medical Subject Heading (MeSH) terms and associated keywords discovered using the MeSH library. (Table 2)

**Table 2:** Search theme and terms.

Domain	Search term
Population	Fibromyalgia, chronic fatigue syndrome
Physical activity promotion strategy	Exercise, Exercise training, Physical activity, Endurance training, Pedometer, accelerometer, Behaviour therapy, theories of PA promotion, Health education, primary prevention
Outcomes	Pain, musculoskeletal pain, chronic pain, fatigue, exercise tolerance, physical endurance, activities of daily living, and quality of life
Study design	Randomized controlled trial OR quasi experimental study OR pre-post study OR before versus after study OR experimental study OR effectiveness study

PA: Physical Activity.

The search strategy, which is likely to encompass a wide range of keywords and regulated vocabulary, will be designed with the support of a university librarian and a subject expert. A search will be created in PubMed/Medline (Table 3) without using any timeframes, and this will be reciprocated for additional specified databases such as Scopus, Web of Science, ProQuest, and CINAHL. The screened studies must meet the following criteria to be included.

**Table 3:** Search Strategy for PubMed/Medline.

Search Strategy #1: ((((((((((((((((fibromyalgia) OR (fibromyalgia[MeSH Terms])) OR (fibromyalgia[Title/Abstract])) OR (fibromyalgia[Title])) OR (fatigue syndrome[Title])) OR (fatigue syndrome[Title/Abstract]))) OR (fatigue syndrome)) OR (chronic fatigue syndrome)) OR (chronic fatigue syndrome[Title])) OR (chronic fatigue syndrome[Title/Abstract])) OR (Primary fibromyalgia[Title/Abstract])) OR (Primary fibromyalgia[Title])) OR (Primary fibromyalgia)) OR (secondary fibromyalgia)) OR (secondary fibromyalgia[Title])) OR (secondary fibromyalgia[Title/Abstract]) #2: ((((((((((((((((((((((((pain[Title/Abstract]) OR (pain[Title])) OR (pain[MeSH Terms])) OR (pain)) OR (musculoskeletal pain)) OR (musculoskeletal pain[Title])) OR (musculoskeletal pain[Title/Abstract])) OR (chronic pain[Title/Abstract])) OR (chronic pain[Title])) OR (chronic pain[MeSH Terms])) OR (chronic pain)) OR (fatigue)) OR (fatigue[Title/Abstract])) OR (fatigue[Title])) OR (fatigue[MeSH Terms])) OR (exercise tolerance[MeSH Terms])) OR (exercise tolerance)) OR (exercise tolerance[Title])) OR (exercise tolerance[Title/Abstract])) OR (activities of daily living[Title/Abstract])) OR (activities of daily living[Title])) OR (activities of daily living)) OR (quality of life)) OR (quality of life[Title/Abstract])) OR (quality of life[Title]) #3: ((((((((((((((((((((((((((((((((((((((((((((((((((exercise[Title]) OR (exercise[Title/Abstract])) OR (exercise[MeSH Terms])) OR (exercise)) OR (physical activity)) OR (physical activity[Title/Abstract])) OR (physical activity[Title])) OR (aerobic exercise[Title])) OR (aerobic exercise[Title/Abstract])) OR (aerobic exercise)) OR (resistance exercise)) OR (resistance exercise[Title])) OR (resistance exercise[Title/Abstract])) OR (health promotion[Title/Abstract])) OR (health promotion[Title])) OR (health promotion)) OR (behavior)) OR (behavior[MeSH Terms])) OR (behavior[Title/Abstract])) OR (behavior[Title])) OR (behaviour therapy[Title])) OR (behaviour therapy[Title/Abstract])) OR (behaviour therapy)) OR (biopsychosocial)) OR (biopsychosocial[Title])) OR (biopsychosocial[Title/Abstract])) OR (pedometer[Title/Abstract])) OR (pedometer[Title])) OR (pedometer)) OR (accelerometer)) OR (accelerometer[Title])) OR (accelerometer[Title/Abstract])) OR (trans theoretical model[Title/Abstract])) OR (trans theoretical model[Title])) OR (trans theoretical model)) OR (social cognitive theory)) OR (social cognitive theory[Title])) OR (social cognitive theory[Title/Abstract])) OR (determination theory[Title/Abstract])) OR (determination theory[Title])) OR (determination theory)) OR (theory of planned behavior)) OR (theory of planned behavior[Title])) OR (theory of planned behavior[Title/Abstract])) OR (maintenance[Title/Abstract])) OR (maintenance[Title])) OR (maintenance)) OR (physical fitness)) OR (physical fitness[MeSH Terms])) OR (physical fitness[Title])) OR (physical fitness[Title/Abstract])

Inclusion criteria are as follows: Firstly, studies must focus on participants with a confirmed diagnosis of FMS, aligning with the objective of understanding PA promotion specifically within this population. Secondly, eligible studies must be published, peer-reviewed articles that employ robust research designs, including randomized controlled trials, quasi-experimental studies, pre-post intervention studies, before-and-after comparisons, experimental studies, or effectiveness studies. These studies should assess various PA promotion strategies. Specifically, we will consider interventions involving walking, Nordic walking, stretching, resistance training, aerobic exercises, yoga, Pilates, behavioral strategies for PA promotion, or educational interventions aimed at enhancing PA engagement. Furthermore, studies that compare any form of exercise intervention against control treatments, standard care, or alternative PA modalities will also be included. Lastly, studies must report on relevant outcome variables, including pain, fatigue, exercise tolerance, and quality of life, to facilitate a comprehensive understanding of the impact of PA promotion on FMS. Conversely, studies will be excluded based on the following criteria: any research involving participants for whom FMS is not the primary diagnosis will be excluded to maintain a focused analysis on this specific condition. Additionally, studies involving participants younger than 18 years will be omitted to ensure that the review focuses on adult populations. We will also exclude non-original research articles, including review articles, case reports, case series, editorials, commentaries, short communications, letters to the editor, systematic reviews, cohort studies, cross-sectional studies, and other scoping reviews, as these do not provide the primary empirical data necessary for our analysis. Finally, full-text studies published in languages other than English will be excluded, as this could pose challenges in accurately interpreting and synthesizing the findings. Through these carefully defined criteria, our review aims to curate a representative and high-quality body of literature that elucidates the role of PA in managing FMS.

**Stage 3: Study selection**

The duplicate studies will be removed, after which it will be imported to Rayyan.ai [15]. The investigators will meet at the review process's starting, intermediate, and final stages to ensure no difficulties or ambiguities are encountered. Three investigators will independently review the title and abstract to determine the eligibility of the studies. The fourth investigator will intervene if any disagreement occurs. The full texts of the included research articles will be assessed in a similar way. Preferred Reporting Items for Systematic Reviews and Meta-Analyses extension for Scoping Reviews (PRISMA-ScR) checklist will be used for reporting the scoping review. (Attached in supplementary material)

**Stage 4: Charting the data**

The extracted data will be charted in a tabular format displaying the key factors of the studies included. This will provide a clear summary of the studies and distinct answers to the precise research questions of the study. Three independent investigators will be involved in charting the data which will be including the publication details like author, year; population characteristics like age, gender, height, weight and BMI, geographical setting; methodological aspects such as study design, sample size, sampling method, intervention, and outcome; the findings of the study with its recommendations. These details will also be depicted in tabular format as mentioned in Table 4.

**Table 4:** Data extraction table.

Author Name (Year of Publication)	Participant Age (in Years)	Gender	Height (in metre)	Weight (in Kg)	BMI	Study Location	Study design	Sampling method	Sample size	Type of intervention	Frequency of Intervention	Primary Outcome	Secondary Outcome	Results	Conclusion
															

															

															

**Quality Assessment:** The critical appraisal instrument developed by Joanna Briggs[16] will be used to critically appraise all the papers included. Two authors will assess the study's quality independently and settle any issues through discussion and mutual agreement. The complete risk assessment will be provided in a tabular or graphical format, with a methodical narrative synthesis to describe it.

**Stage 5. Collating, summarizing, and reporting the results**

This stage will be carried out in three distinct steps. An analysis including descriptive narrative summary analysis of PA and FMS will be carried out, using content analysis. The results will be reported with feasible outcomes referring to the research question. Results and key findings will be presented in a descriptive form with figures and tables. After the results have been summarised in presentable data format, the rationale of the findings will be discussed in relation to the study objectives. The inferences of the analysis will be assessed for understanding its implications on future practices and protocols for FMS as well as scope for further research.

## Protocol validation

Fibromyalgia is a chronic, non-inflammatory musculoskeletal condition disease manifested with widespread pain, stiffness, and impairment to activities of daily living. The results of the present review will provide an in-depth knowledge regarding the available literature and approaches for promoting PA in FMS individuals. The synthesis of available literature and its mapping will provide invaluable guidance for exercise professionals to understand available PA promotional strategy for fibromyalgia syndrome.

## Limitations

Not applicable.

## CRediT authorship contribution statement

**Saumya Srivastava:** Conceptualization, Methodology, Software, Writing – original draft. **Aishwarya Nair:** Conceptualization, Methodology, Software, Writing – original draft. **Dhanesh Kumar K U:** Conceptualization, Methodology, Validation, Data curation, Writing – original draft, Supervision, Writing – review & editing. **Rakesh Krishna Kovela:** Conceptualization, Methodology, Software, Writing – original draft. **Vaishali K:** Conceptualization, Methodology, Validation, Data curation, Writing – original draft, Supervision, Writing – review & editing. **Ashwani Verma:** Conceptualization, Methodology, Software, Writing – original draft. **Mukesh Kumar Sinha:** Conceptualization, Methodology, Validation, Data curation, Writing – original draft, Supervision, Writing – review & editing.

## Declaration of Competing Interest

The authors declare that they have no known competing financial interests or personal relationships that could have appeared to influence the work reported in this paper.

## Data Availability

No data was used for the research described in the article.
